# Prevalence and characterization of human *mecC* methicillin-resistant *Staphylococcus aureus* isolates in England

**DOI:** 10.1093/jac/dkt462

**Published:** 2013-11-27

**Authors:** G. K. Paterson, F. J. E. Morgan, E. M. Harrison, E. J. P. Cartwright, M. E. Török, R. N. Zadoks, J. Parkhill, S. J. Peacock, M. A. Holmes

**Affiliations:** 1 Department of Veterinary Medicine, University of Cambridge, Madingley Road, Cambridge CB3 0ES, UK; 2 Department of Medicine, University of Cambridge, Addenbrooke's Hospital, Cambridge CB2 0SP, UK; 3 Cambridge Microbiology and Public Health Laboratory, Public Health England, Cambridge, UK; 4 Cambridge University Hospitals NHS Foundation Trust, Cambridge, UK; 5 University of Glasgow, Institute of Biodiversity, Animal Health and Comparative Medicine, Glasgow, UK; 6 Moredun Research Institute, Penicuik, UK; 7 The Wellcome Trust Sanger Institute, Wellcome Trust, Genome Campus, Cambridge CB10 1SA, UK

**Keywords:** MRSA, *mec* genes, *S. aureus*, surveillance

## Abstract

**Objectives:**

There are limited data available on the epidemiology and prevalence of methicillin-resistant *Staphylococcus aureus* (MRSA) in the human population that encode the recently described *mecA* homologue, *mecC*. To address this knowledge gap we undertook a prospective prevalence study in England to determine the prevalence of *mecC* among MRSA isolates.

**Patients and methods:**

Three hundred and thirty-five sequential MRSA isolates from individual patients were collected from each of six clinical microbiology laboratories in England during 2011–12. These were tested by PCR or genome sequencing to differentiate those encoding *mecA* and *mecC. mecC*-positive isolates were further characterized by multilocus sequence typing, *spa* typing, antimicrobial susceptibility profile and detection of PBP2a using commercially available kits.

**Results:**

Nine out of the 2010 MRSA isolates tested were *mecC* positive, indicating a prevalence among MRSA in England of 0.45% (95% CI 0.24%–0.85%). The remainder were *mecA* positive. Eight out of these nine *mecC* MRSA isolates belonged to clonal complex 130, the other being sequence type 425. Resistance to non-β-lactam antibiotics was rare among these *mecC* MRSA isolates and all were phenotypically identified as MRSA using oxacillin and cefoxitin according to BSAC disc diffusion methodology. However, all nine *mecC* isolates gave a negative result using three different commercial PBP2a detection assays.

**Conclusions:**

*mecC* MRSA are currently rare among MRSA isolated from humans in England and this study provides an important baseline prevalence rate to monitor future changes, which may be important given the increasing prevalence of *mecC* MRSA reported in Denmark.

## Introduction

*Staphylococcus aureus* is a versatile, opportunistic pathogen able to cause a wide range of diseases in humans, from minor skin infections to severe illnesses such as septicaemia, toxic shock, endocarditis and pneumonia. It is also able to colonize and infect a variety of other host species, including farm and companion animals and wildlife. The emergence and dissemination of methicillin-resistant *S. aureus* (MRSA) since the early 1960s has posed a major challenge to the treatment of *S. aureus* infections. Methicillin resistance in *S. aureus* is conferred by the acquisition of one of several staphylococcal cassette chromosome *mec* (SCC*mec*) elements, which carry the *mecA* gene encoding a penicillin-binding protein homologue (PBP2a) with reduced affinity for β-lactam antibiotics.^1^ We identified a novel *mecA* homologue, *mecA*_LGA251_, encoded in a new SCC*mec* element, designated type XI, among human and bovine MRSA isolates in the UK and Denmark.^2^ This *mecA* homologue, subsequently named *mecC*,^1^ exhibits only 69% identity at the DNA level and 63% identity at the protein level to the previously described *mecA*/PBP2a. As a result, it is not detectable by routine *mecA*-specific PCR approaches or PBP2a slide agglutination tests. *mecC* MRSA have now been isolated in small numbers from humans and a wide range of other host species in several European countries: Republic of Ireland,^3^ France,^4^ Sweden,^5–7^ the Netherlands,^8^ Germany,^8–11^ Austria,^12^ Switzerland,^13^ Finland,^14^ Spain,^15^ Norway^16^ and Belgium.^17,18^ However, the origin and epidemiology of these strains are poorly understood and there are limited data on their prevalence. Importantly, the frequency of *mecC* MRSA has increased significantly in Denmark since 2003.^19^

To provide baseline data for future surveillance in the UK, we undertook a prospective survey of a total of 2010 MRSA isolates collected from six clinical microbiology laboratories in England and screened these by PCR or genome sequencing for *mecA* and *mecC*.

## Methods

### Isolate collection and assessment of mec gene status

Three hundred and thirty-five sequential MRSA isolates from individual patients were identified according to local procedures from screening and clinical samples at five hospital clinical microbiology laboratories from October 2011 to August 2012 (Table 1). These were sent to Cambridge for PCR detection of *mecA* and *mecC*, as described previously.^17^ These were isolates drawn from hospitals and other healthcare providers in the catchment area of each laboratory, including community-based general practitioners. Methicillin resistance was based on phenotypic resistance (cefoxitin disc diffusion, Vitek 2 or chromogenic agars) in all cases and not on molecular detection of *mecA* or PBP2a. Isolates from a sixth hospital (Addenbrooke's Hospital, Cambridge; Table 1) were collected as above and genome sequenced as part of an independent study. These were not assessed by PCR but by interrogation of their genome sequences using BLAST analysis to identify *mecA* and *mecC* MRSA isolates with confirmation of the presence of *femB* as a species marker of *S. aureus*. The analysis of 2010 isolates provides the power to detect *mecC* MRSA prevalence at a lower limit of 0.05% at the 95% confidence level.

**Table 1.** Contributing hospitals

Clinical microbiology laboratory	Location (city and county)	First sample date	Last sample date	Number of *mecA*:*mecC* MRSA
Royal Preston Hospital	Preston, Lancashire	October 2011	June 2012	335 : 0
Countess of Chester Hospital	Chester, Cheshire	November 2011	August 2012	335 : 0
Nottingham Universities Hospitals	Nottingham, Nottinghamshire	January 2012	May 2012	333 : 2
Musgrove Hospital	Taunton, Somerset	November 2011	May 2012	333 : 2
Royal Cornwall Hospitals Trust	Truro, Cornwall	November 2011	July 2012	333 : 2
Addenbrooke's Hospital	Cambridge, Cambridgeshire	April 2012	June 2012	332 : 3

### Antimicrobial susceptibility testing and slide agglutination for PBP2a

All *mecC* MRSA isolates were analysed using the Vitek 2 system (bioMérieux, Basingstoke, UK). In brief, suspensions of cultures were made in 0.45% sodium solution from growth on Columbia blood agar, adjusted to a turbidity equivalent to that of a 0.5 McFarland standard and used to load the test cards, which were used in accordance with the manufacturer's instructions. The Staph AST-P620 card was automatically filled, sealed and inserted into the Vitek 2 reader–incubator module (incubation temperature 37°C), and fluorescence measurements were performed every 15 min for up to 18 h. Cefoxitin and oxacillin resistances were also assayed by disc diffusion following BSAC guidelines (version 11.1 May 2012) and the MICs of cefoxitin and oxacillin were determined using Etest strips (bioMérieux). All *mecC* MRSA isolates identified were tested with three commercially available PBP2a detection assays according to the manufacturers' instructions: the Mastalex™ MRSA Test (Mast Diagnostics, Bootle, UK), the Penicillin Binding Protein (PBP2′) Latex Agglutination Test (Oxoid, Basingstoke, UK) and the Alere™ PBP2a Culture Colony Test (Alere Ltd, Stockport, UK). The *mecA*-positive MRSA strain NCTC12493 was used as a positive control.

### Genome sequencing and spa typing

All *mecC* MRSA isolates underwent whole genome sequencing using the HiSeq2000 platform (Illumina, Little Chesterford, UK) to confirm their *mecC* gene status and determine their multilocus sequence type (ST). Isolates that were PCR negative for either *mecA* or *mecC* were also genome sequenced to confirm their *mec* gene status. The species identity of isolates negative by PCR for *femB* was tested by assessing their growth and morphology on Staph Brilliance 24 and MRSA Brilliance 2 agar plates (both Oxoid) and by PCR to detect *nuc*.^20^ *spa* typing was performed using the primers spa-1113f (5′-TAA AGA CGA TCC TTC GGT GAG C-3′) and spa-1514r (5′-CAG CAG TAG TGC CGT TTG CTT-3′) as described by Ridom GmbH (Würzburg, Germany).

## Results and discussion

PCR (or genome sequence analysis in the case of Addenbrooke's Hospital, Cambridge) revealed that 9 isolates out of a total of 2010 MRSA collected were *mecC* MRSA. These *mecC* MRSA isolates were largely from screening samples (six isolates), but included three isolates from skin and soft tissue infections. The remaining MRSA isolates were all *mecA* positive, which provides a prevalence rate of *mecC* MRSA among all MRSA collected of 0.45% with a 95% CI of 0.24%–0.85%. All 2010 isolates were confirmed to be *S. aureus*. In the majority of cases this identification was based on the presence of *femB* as detected by PCR or genome sequencing. However, 12 out of the 1675 isolates (0.72%) tested by PCR for *femB* were negative for an amplicon and were instead confirmed to be *S. aureus* based on their growth on Staph Brilliance and MRSA Brilliance agar plates and all were positive for *nuc*. The basis for the negative *femB* PCR result is under investigation and may relate to divergence in the *femB* primer binding sites. Indeed, a small number of *S. aureus* isolates negative for *femB* using alternative PCR approaches have been reported previously.^21,22^ Two isolates were negative by PCR for both *mecA* and *mecC*, but genome sequencing revealed that they were indeed *mecA* positive and carried previously described *mecA* genes (NCBI accession numbers FJ390057 and AF411935) with divergence in the primer binding sites used in this study.

Genome sequencing confirmed that each isolate positive for *mecC* by PCR encoded *mecC* within an SCC*mec* type XI. Multilocus ST derived from the genome sequences revealed five different STs among the nine isolates, including a novel ST, ST2574. Eight of the isolates belonged to clonal complex (CC) 130, with the remaining isolate belonging to ST425. Five *spa* types were represented: t843, t6220, t9280, t11702 and t11706 (Table 2). All *mecC* MRSA isolates were resistant to cefoxitin and oxacillin using BSAC guidelines for disc diffusion, while MICs varied from 8 to 32 mg/L for oxacillin and from 8 to 16 mg/L for cefoxitin (Table 2). Antimicrobial susceptibility testing using Vitek 2 revealed that resistance to non-β-lactam antibiotics was rare, the only example being a single isolate, Ta222, displaying resistance to erythromycin and inducible resistance to clindamycin (Table 2). All nine isolates displayed the unusual Vitek 2 resistance profile of being resistant to cefoxitin, but susceptible to oxacillin. This feature of *mecC* MRSA, likely caused by structural differences between the *mecA*- and *mecC*-encoded PBP2a,^23^ has been described previously and may be helpful in the identification of *mecC* MRSA isolates.^24^ The susceptibility to oxacillin seen using Vitek 2 is in disagreement with our oxacillin disc diffusion results. *mecC*-encoded PBP2a has been shown to be less stable at 37°C than at 30°C,^23^ which may explain this discrepancy, oxacillin disc diffusion being performed at 30°C, but Vitek 2 analysis at 37°C. Cefoxitin resistance is presumably still seen using Vitek 2, even at 37°C, because of the higher affinity *mecC*-encoded PBP2a has for cefoxitin versus oxacillin.^23^ All nine *mecC* MRSA isolates gave negative results when assayed with three different commercial PBP2a slide agglutination assays, confirming the difficulty of detecting *mecC* MRSA using this approach.

**Table 2.** Characteristics of *mecC* MRSA isolates

Isolate	Hospital	ST	CC	*spa* type	Vitek profile^a^	Oxacillin MIC (mg/L)	Cefoxitin MIC (mg/L)	Site of isolation
N35	Nottingham	130	130	t843	benzylpenicillin, cefoxitin	16	16	leg ulcer
N147	Nottingham	130	130	t11702	benzylpenicillin, cefoxitin	24	8	wound swab
Tr8	Truro	2573	130	t843	benzylpenicillin, cefoxitin	16	12	multisite screen
Tr34	Truro	1245	130	t11706	benzylpenicillin, cefoxitin	32	8	multisite screen
Ta222	Taunton	425	425	t11706	benzylpenicillin, cefoxitin, erythromycin, inducible resistance to clindamycin	8	8	groin screen
Ta320	Taunton	1245	130	t6220	benzylpenicillin, cefoxitin	32	12	toe wound
Ca155	Cambridge	1245	130	t6220	benzylpenicillin, cefoxitin	24	12	multisite screen
Ca226	Cambridge	2574 (new)	130	t9280	benzylpenicillin, cefoxitin	16	8	multisite screen
Ca322	Cambridge	1245	130	t843	benzylpenicillin, cefoxitin	32	12	multisite screen

^a^Only resistances are shown. Resistance to benzylpenicillin, cefoxitin, oxacillin, ciprofloxacin, erythromycin, chloramphenicol, daptomycin, fusidic acid, gentamicin, linezolid, mupirocin, nitrofurantoin, rifampicin, teicoplanin, tetracycline, tigecycline, trimethoprim, vancomycin and clindamycin was tested for, as well as inducible resistance to clindamycin.

This is the first formal prospective prevalence study of *mecC* MRSA performed in the UK and these data provide a baseline prevalence for the future surveillance of *mecC* MRSA in England. Continued monitoring of *mecC* is potentially important given the increase in prevalence of *mecC* MRSA reported in Denmark.^19^ There are few other data on *mecC* MRSA prevalence elsewhere, but in Germany a large multicentre prospective study identified a single *mecC* isolate among 1604 tested in 2004–05 and again a single isolate from 1603 tested in 2010–11.^10^ This indicates a prevalence of 0.06% with no change between the study periods. In contrast, the prevalence in Denmark was both higher and increasing, rising from 1.91% in 2010 to 2.78% in 2011.^19^ A survey of 565 human MRSA isolates in Switzerland failed to find any *mecC* MRSA, indicating that the prevalence there is lower than in Denmark.^13^ Clearly, there are significant and as yet unexplained differences in *mecC* MRSA prevalence between different countries, and the recent increase reported in Denmark suggests that it would be prudent to monitor prevalence in the UK and elsewhere.

None of the hospitals used oxacillin to identify MRSA, which has been shown to be less reliable than cefoxitin for the detection of *mecC* MRSA.^25^ Nonetheless, it is possible that some *mecC* MRSA may have been missed during primary isolation. For instance, small numbers of *mecC* MRSA isolates grow poorly on MRSA-selective agars,^9,17^ presumably due to their having low cefoxitin/oxacillin MIC values. An area for future study may be the comparison and standardization of primary isolation methods in relation to *mecC* MRSA.

The majority of *mecC* MRSA isolates found in our survey belonged to CC130, which agrees with the data of Garcia-Alvarez *et al*.^2^ showing that CC130 was the most common lineage among their retrospective testing for *mecC* MRSA among human isolates in the UK and Denmark. Both CC130 and ST425 are the predominant lineages among *mecC* MRSA isolates found not only in humans but also in other host species elsewhere, and genome sequencing has provided strong evidence of cross-species transmission of *mecC* MRSA between humans and livestock.^26^ Of the five *spa* types recovered in this study, neither t11702 nor t11706 appear to have been reported previously among *mecC* MRSA, whilst the other three, t843, t6220 and t9280, have.^2,27^ There were multiple CCs belonging to the same *spa* type and multiple *spa* types within the same CC, illustrating the difficulty of inferring CC from *spa* type data.

As reported for *mecC* MRSA isolated elsewhere in Europe and from different host species,^9,11,15,17,27^ resistance to non-β lactam antibiotics was uncommon among these English *mecC* MRSA isolates.

The origins of *mecC* MRSA and SCC*mec* type XI are unclear, but *mecC* has also been detected in *Staphylococcus stepanovicii*,^12^ *Staphylococcus xylosus*^28^ and *Staphylococcus sciuri*.^29^ This suggests a possible origin for *mecC* in coagulase-negative staphylococci, as proposed for *mecA*,^30,31^ and clinical microbiology laboratories should therefore be aware not only of *mecC* MRSA but of the possible occurrence of *mecC* in other pathogenic species of methicillin-resistant staphylococci.

## Funding

This work was supported by a Medical Research Council Partnership Grant (G1001787/1) held between the Department of Veterinary Medicine, University of Cambridge (M. A. H.), the School of Clinical Medicine, University of Cambridge (S. J. P.), the Moredun Research Institute (R. N. Z.) and the Wellcome Trust Sanger Institute (J. P. and S. J. P.).

## Transparency declarations

Competing interests: none to declare.

The funder had no role in the study design, data collection, analysis, decision to publish, or preparation of the manuscript.

## References

[1] Ito T, Hiramatsu K, Tomasz A (2012). Guidelines for reporting novel *mecA* gene homologues. Antimicrob Agents Chemother.

[2] Garcia-Alvarez L, Holden MTG, Lindsay H (2011). Meticillin-resistant *Staphylococcus aureus* with a novel *mecA* homologue in human and bovine populations in the UK and Denmark: a descriptive study. Lancet Infect Dis.

[3] Shore AC, Deasy EC, Slickers P (2011). Detection of staphylococcal cassette chromosome *mec* type XI carrying highly divergent *mecA*, *mecI*, *mecR1*, *blaZ*, and *ccr* genes in human clinical isolates of clonal complex 130 methicillin-resistant *Staphylococcus aureus*. Antimicrob Agents Chemother.

[4] Laurent F, Chardon H, Haenni M (2012). MRSA harboring *mecA* variant gene *mecC*, France. Emerg Infect Dis.

[5] Hellman J, Olsson-Liljequist B (2012). A Report on Swedish Antibiotic Utilisation and Resistance in Human Medicine.

[6] Unnerstad HE, Bengtsson B, Rantzien MH (2013). Methicillin-resistant *Staphylococcus aureus* containing *mecC* in Swedish dairy cows. Acta Vet Scand.

[7] Monecke S, Gavier-Widen D, Mattsson R (2013). Detection of *mecC*-positive *Staphylococcus aureus* (CC130-MRSA-XI) in diseased European hedgehogs (*Erinaceus europaeus*) in Sweden. PLoS One.

[8] Sabat AJ, Koksal M, Akkerboom V (2012). Detection of new methicillin-resistant *Staphylococcus aureus* strains that carry a novel genetic homologue and important virulence determinants. J Clin Microbiol.

[9] Cuny C, Layer F, Strommenger B (2011). Rare occurrence of methicillin-resistant *Staphylococcus aureus* CC130 with a novel *mecA* homologue in humans in Germany. PLoS One.

[10] Schaumburg F, Koeck R, Mellmann A (2012). Population dynamics among methicillin-resistant *Staphylococcus aureus* isolates in Germany during a 6-year period. J Clin Microbiol.

[11] Walther B, Wieler LH, Vincze S (2012). MRSA variant in companion animals. Emerg Infect Dis.

[12] Loncaric I, Kubber-Heiss A, Posautz A (2013). Characterization of methicillin-resistant *Staphylococcus* spp. carrying the *mecC* gene, isolated from wildlife. J Antimicrob Chemother.

[13] Basset P, Prod'hom G, Senn L (2013). Very low prevalence of meticillin-resistant *Staphylococcus aureus* carrying the *mecC* gene in western Switzerland. J Hosp Infect.

[14] Gindonis V, Taponen S, Myllyniemi AL (2013). Occurrence and characterization of methicillin-resistant staphylococci from bovine mastitis milk samples in Finland. Acta Vet Scand.

[15] Garcia-Garrote F, Cercenado E, Marin M (2014). Methicillin-resistant *Staphylococcus aureus* carrying the *mecC* gene: emergence in Spain and report of a fatal case of bacteraemia. J Antimicrob Chemother.

[16] Medhus A, Slettemeas JS, Marstein L (2013). Methicillin-resistant *Staphylococcus aureus* with the novel *mecC* gene variant isolated from a cat suffering from chronic conjunctivitis. J Antimicrob Chemother.

[17] Paterson GK, Larsen AR, Robb A (2012). The newly described *mecA* homologue, *mecA*_LGA251_, is present in methicillin-resistant *Staphylococcus aureus* isolates from a diverse range of host species. J Antimicrob Chemother.

[18] Vandendriessche S, Vanderhaeghen W, Soares FV (2013). Prevalence, risk factors and genetic diversity of methicillin-resistant *Staphylococcus aureus* carried by humans and animals across livestock production sectors. J Antimicrob Chemother.

[19] Petersen A, Stegger M, Heltberg O (2013). Epidemiology of methicillin-resistant *Staphylococcus aureus* carrying the novel *mecC* gene in Denmark corroborates a zoonotic reservoir with transmission to humans. Clin Microbiol Infect.

[20] Brakstad OG, Aasbakk K, Maeland JA (1992). Detection of *Staphylococcus aureus* by polymerase chain reaction amplification of the *nuc* gene. J Clin Microbiol.

[21] Mohanasoundaram KM, Lalitha MK (2008). Comparison of phenotypic versus genotypic methods in the detection of methicillin resistance in *Staphylococcus aureus*. Indian J Med Res.

[22] Kobayashi N, Wu H, Kojima K (1994). Detection of *mecA*, *femA*, and *femB* genes in clinical strains of staphylococci using polymerase chain reaction. Epidemiol Infect.

[23] Kim C, Milheirico C, Gardete S (2012). Properties of a novel PBP2A protein homolog from *Staphylococcus aureus* strain LGA251 and its contribution to the β-lactam-resistant phenotype. J Biol Chem.

[24] Cartwright EJP, Paterson GK, Raven KE (2013). Use of Vitek 2 antimicrobial susceptibility profile to identify *mecC* in methicillin-resistant *Staphylococcus aureus*. J Clin Microbiol.

[25] Skov R, Larsen AR, Kearns A (2014). Phenotypic detection of *mecC*-MRSA: cefoxitin is more reliable than oxacillin. J Antimicrob Chemother.

[26] Harrison EM, Paterson GK, Holden MTG (2013). Whole genome sequencing identifies zoonotic transmission of MRSA isolates with the novel *mecA* homologue *mecC*. EMBO Mol Med.

[27] Barraud O, Laurent F, François B (2013). Severe human bone infection due to methicillin-resistant *Staphylococcus aureus* carrying the novel *mecC* variant. J Antimicrob Chemother.

[28] Harrison EM, Paterson GK, Holden MTG (2013). A *Staphylococcus xylosus* isolate with a new *mecC* allotype. Antimicrob Agents Chemother.

[29] Harrison EM, Paterson GK, Holden MTG (2014). A novel hybrid SCC*mec*-*mecC* region in *Staphylococcus sciuri*. J Antimicrob Chemother.

[30] Couto I, de Lencastre H, Severina E (1996). Ubiquitous presence of a *mecA* homologue in natural isolates of *Staphylococcus sciuri*. Microb Drug Resist.

[31] Tsubakishita S, Kuwahara-Arai K, Sasaki T (2010). Origin and molecular evolution of the determinant of methicillin resistance in staphylococci. Antimicrob Agents Chemother.

